# The anti-inflammatory and immunosuppressive effects of glucocorticoids, recent developments and mechanistic insights

**DOI:** 10.1016/j.mce.2010.04.005

**Published:** 2011-03-15

**Authors:** Agnes E. Coutinho, Karen E. Chapman

**Affiliations:** Endocrinology Unit, Centre for Cardiovascular Sciences, The Queen's Medical Research Institute, University of Edinburgh, 47 Little France Crescent, Edinburgh, EH16 4TJ, UK

**Keywords:** Glucocorticoid, Inflammation, 11β-Hydroxysteroid dehydrogenase

## Abstract

Since the discovery of glucocorticoids in the 1940s and the recognition of their anti-inflammatory effects, they have been amongst the most widely used and effective treatments to control inflammatory and autoimmune diseases. However, their clinical efficacy is compromised by the metabolic effects of long-term treatment, which include osteoporosis, hypertension, dyslipidaemia and insulin resistance/type 2 diabetes mellitus. In recent years, a great deal of effort has been invested in identifying compounds that separate the beneficial anti-inflammatory effects from the adverse metabolic effects of glucocorticoids, with limited effect. It is clear that for these efforts to be effective, a greater understanding is required of the mechanisms by which glucocorticoids exert their anti-inflammatory and immunosuppressive actions. Recent research is shedding new light on some of these mechanisms and has produced some surprising new findings. Some of these recent developments are reviewed here.

## 1 Introduction

Natural and synthetic glucocorticoids remain at the forefront of anti-inflammatory and immunosuppressive therapies. They are widely used to treat both acute and chronic inflammations, including rheumatoid arthritis, inflammatory bowel disease, multiple sclerosis, psoriasis and eczema, as well as being used in treatment of certain leukaemias and in immunosuppressive regimes following organ transplant. At any one time, an estimated ∼1% of the total adult population of the UK receives oral glucocorticoid therapy (van Staa et al., 2000). However, long-term use of oral glucocorticoids is associated with serious side effects, including osteoporosis, metabolic disease and increased risk of cardiovascular disease (Wei et al., 2004; Souverein et al., 2004; de Vries et al., 2007; Vegiopoulos and Herzig, 2007)—in themselves, somewhat paradoxically, inflammatory conditions. Over the 60 years since the discovery of glucocorticoids, much has been learnt of the molecular mechanisms by which they act (recently reviewed; Perretti and Ahluwalia (2000), Necela and Cidlowski (2004), Yeager et al. (2004), Rhen and Cidlowski (2005), Smoak and Cidlowski (2004), Tuckermann et al. (2005), Kleiman and Tuckermann (2007), Newton and Holden (2007) and De Bosscher and Haegeman (2009)), with milestones being the characterisation of the glucocorticoid receptor (GR) as a DNA binding protein that regulates transcription initiation (reviewed, Yamamoto (1985)), the cloning of GR (Hollenberg et al., 1985; Miesfeld et al., 1986) and the discovery that many of the immunosuppressive actions of glucocorticoids are mediated by interference with signalling by the key inflammatory transcriptional regulators; NF-κB and AP-1 (Jonat et al., 1990; Yangyen et al., 1990; Heck et al., 1994) (reviewed, McKay and Cidlowski (1999)). However, much also remains unknown and the past few years have provided crucial mechanistic insights into the dynamic nature of GR interactions with DNA and higher order chromatin structures, into the subtleties of ligand effects on GR function, the regulation of endogenous ligand access to GR, glucocorticoid effects on leukocyte differentiation and function and the mechanistic basis for some of the repressive actions of glucocorticoids, critical for their anti-inflammatory effects.

## 2 Inflammation and its resolution

The host inflammatory response is a primary defence mechanism engaged immediately following injury or infection which is necessary to restore homeostasis following successful elimination of the injurious agent, ultimately leading to resolution and tissue repair. Although categorically distinct, the innate (the relatively non-specific immediate host defence system that provides a rapid reaction to infection and tissue damage) and adaptive (the more slowly acquired, highly antigen-specific response) immune systems interact and often overlap during an inflammatory response. Indeed, although acute inflammation is largely mediated by the innate immune system, the adaptive immune system often plays a major role in chronic inflammatory disease, with dysregulated lymphocyte responses.

Inflammation is initiated at the site of injury by resident cells, particularly mast cells and resident macrophages, which release pro-inflammatory mediators including bioactive amines, lipid mediators and cytokines—typically TNF-α and IL-1. These cause vasodilation, increased capillary permeability (humoral response) and leukocyte emigration into injured tissues (cellular response), resulting in the hallmark pain, heat, redness and swelling of inflammation as well as generating a chemotactic gradient to guide and activate recruited cells to the site of injury. Although specific characteristics depend on the immune exposure (e.g. irritant *vs* pathogen), the recruitment process and activation of inflammatory cells are common. Activated granulocytes, crucial to contain microbial infection, are rapidly attracted to the inflamed site, followed by monocyte emigration from blood vessels and subsequent maturation into macrophages. Once at the inflamed site, neutrophils undergo constitutive apoptosis, functionally isolating them from the inflammatory environment by loss of stimulated chemotaxis, phagocytosis, degranulation and respiratory burst (Haslett et al., 1991; Whyte et al., 1993), whilst at the same time, facilitating safe removal of their potentially histotoxic contents by macrophages (Savill et al., 2002). Foreign antigens are taken up by antigen presenting cells; particularly dendritic cells, but also macrophages, that then migrate to draining lymph nodes where they instruct the adaptive immune system (T and B lymphocytes), shaping the subsequent immune response. As the inflammatory response progresses and evolves, mononuclear cells predominate and resolution normally ensues. Successful resolution of acute inflammation is an active and highly regulated process and dependent on mechanisms engaged early in the inflammatory response that programme the trajectory and form of the subsequent resolution (reviewed Savill et al. (2002), Gilroy (2004) and Serhan and Savill (2005)). Persistence of the initiating stimulus invariably leads to chronic inflammation, with the typical dysregulation between destructive inflammatory and excessive healing responses seen in diseases such as arthritis, atherosclerosis and asthma.

## 3 Glucocorticoids and inflammation

Glucocorticoids inhibit many of the initial events in an inflammatory response. They also promote the resolution of inflammation although the mechanisms by which they do so have received less attention than those associated with suppression of the initial response. Acutely, glucocorticoids inhibit the vasodilation and increased vascular permeability that occurs following inflammatory insult and they decrease leukocyte emigration into inflamed sites, effects that require new protein synthesis (reviewed Perretti and Ahluwalia (2000)). They also alter leukocyte distribution/trafficking (McEwen et al., 1997), death/survival (McEwen et al., 1997; Ashwell et al., 2000; Planey and Litwack, 2000; Herold et al., 2006; McColl et al., 2007) and, importantly, alter cellular differentiation programmes, thus shaping the subsequent response (see below).

Most of the anti-inflammatory and immunosuppressive actions of glucocorticoids are attributable either directly or indirectly to the transcriptional effects of GR agonism which alters transcription of numerous genes in leukocytes, both up and down (Ashwell et al., 2000; McEwen et al., 1997). Although expression of mineralocorticoid receptor (MR), which binds glucocorticoids with high affinity in the absence of 11β-hydroxysteroid dehydrogenase type 2 (11β-HSD2; see below), has been reported in immune cells (Miller et al., 1990; Barish et al., 2005; Lim et al., 2007) (which do not normally express 11β-HSD2 Gilmour et al. (2006), Lim et al. (2007)), it appears of little consequence to the anti-inflammatory effects of glucocorticoids, and may even have pro-inflammatory effects (Lim et al., 2007; Harizi et al., 2008). It should also be noted that some anti-inflammatory effects of glucocorticoids are apparent within minutes and a number are independent of the transcriptional effects of GR (see for example Croxtall et al. (2002), Limbourg et al. (2002) and Stellato (2004)). Notably, glucocorticoids repress transcription of many genes encoding pro-inflammatory cytokines and chemokines, cell adhesion molecules and key enzymes involved in the initiation and/or maintenance of the host inflammatory response (reviewed Barnes (1998), Perretti and Ahluwalia (2000) and Smoak and Cidlowski (2004)). Many of these genes are commonly over-expressed during chronic non-resolving inflammation. Intriguingly, recent data have shown that whereas glucocorticoid administration 1 h following endotoxin (lipopolysaccharide, LPS) challenge is immunosuppressive, administration of the same glucocorticoid dose *prior* to LPS challenge *augments* immune responses (Frank et al., 2010). This was true in both the brain and the liver, raising the possibility that chronic hypothalamic–pituitary–adrenal (HPA) axis activation (as may occur during chronic inflammation for example) exaccerbates inflammation in the brain and elsewhere (reviewed in Sorrells et al. (2009)).

Until recently, it was widely believed that the repressive, anti-inflammatory effects of glucocorticoids were dependent on the ability of GR to inhibit the activity of crucial transcriptional regulators of pro-inflammatory genes, including NF-κB and AP-1, by a mechanism termed “transrepression”. This contrasted with the metabolic actions of glucocorticoids which require gene activation by GR. This view has recently been revised, with the discovery that key anti-inflammatory actions of glucocorticoids are brought about through gene activation (Clark, 2007) (and see below).

## 4 The repressive actions of glucocorticoids

The ability of GR to repress the activity of NF-κB and AP-1 as well as other key immunomodulatory transcription factors has been a major focus of research into the mechanisms underlying the anti-inflammatory effects of glucocorticoids. Although a role for transactivation by GR in repression of NF-κB was implicated – inducing expression of the NF-κB inhibitor, IκBα (Auphan et al., 1995; Scheinman et al., 1995) – this was restricted to certain cell types and did not appear to be a universal mechanism (Heck et al., 1997; Wissink et al., 1998). Biochemical and genetic evidence suggested that negative regulation by a transrepression (or “tethering”) mechanism independent of DNA binding by GR underpinned the repressive effects of glucocorticoids (reviewed Smoak and Cidlowski (2004) and Clark (2007)) and this, by extension, was thought to be responsible for their clinical efficacy. In contrast to gene activation by GR, which at the time was believed to occur exclusively through homodimers of GR binding to palindromic glucocorticoid response elements (GREs) (Beato et al., 1996; Karin, 1998), transrepression involves direct protein-protein binding of GR to other transcription factors and interference with their mechanism of action. The interaction requires the DNA binding domain of GR (but not DNA binding *per se*) and does not require conventional homodimerisation of GR (Barnes, 1998; Karin, 1998; Smoak and Cidlowski, 2004). Transrepression was not affected by mutation of alanine 458 to threonine (A458T) within the dimerisation interface of the DNA binding domain that dramatically reduces GR transactivation of the MMTV-LTR promoter and the tyrosine aminotransferase (TAT) gene promoter, both classically activated by GR through well-characterised palindromic GREs (Payvar et al., 1983; Grange et al., 1989). Considerable support for the transrepression hypothesis came from *in vivo* experiments in mice in which the wild-type GR was replaced by the dimerisation (*dim*) mutant (*GR*^*dim*^ mice) (Reichardt et al., 1998). Glucocorticoid-mediated repression of AP-1-dependent transcription and other pro-inflammatory genes was intact in these mice, in contrast to defective GRE-dependent transactivation (Reichardt et al., 1998, 2001). However, transactivation of some genes, including that encoding phenylethanolamine N-methyltrasferase (PNMT), remained intact (Reichardt et al., 1998). It has since emerged that the GRE in the PNMT gene belongs to a different class of GRE to the palindromic consensus, and comprises a series of repeated half sites to which GR directly binds to activate transcription from the PNMT promoter (Adams et al., 2003). Dual specificity phosphatase (DUSP) 1, also known as MKP-1 (mitogen activated protein kinase [MAPK] phosphatase 1), a crucial anti-inflammatory gene (Hammer et al., 2006; Salojin et al., 2006; Zhao et al., 2006; Maier et al., 2007), also remains fully inducible in *GR*^*dim*^ mice (Abraham et al., 2006), offering an alternative interpretation of their phenotype. Glucocorticoid induction of DUSP1 may have some similarity to that of PNMT, as recent work has shown the glucocorticoid responsive region in the *DUSP1* gene contains 3 closely spaced half sites (Tchen et al., 2009), although a tethering mechanism of activation dependent on C/EBPβ has also been implicated (Johansson Haque et al., 2008). Thus, at least some, but not all, of the dimerisation-independent repressive actions of glucocorticoids may be wholly or partly dependent on induction of DUSP1. Consistent with this, glucocorticoids failed to suppress zymosan-induced inflammation in *DUSP1*^−/−^ mice, whereas inflammation was suppressed in control mice (Abraham et al., 2006). However, *DUSP1*^−/−^ mice remain sensitive to the suppressive effects of glucocorticoids in mast cell-dependent anaphylaxis (Maier et al., 2007), implicating other mechanisms in the anti-inflammatory action of glucocorticoids in this model of inflammation. Intriguingly, *DUSP1*^−/−^ mice are resistant to diet-induced obesity (Wu et al., 2006), raising the possibility that DUSP1 may also be involved in some of the metabolic effects of glucocorticoids.

The anti-inflammatory actions of glucocorticoid-induced genes have been recently reviewed (Clark, 2007). Briefly, as well as DUSP1 and IκB, this class of genes includes IL-10, a potent immunomodulatory and anti-inflammatory cytokine (Couper et al., 2008), Glucocorticoid-induced leucine zipper (GILZ), a protein whose mechanism of action is unclear but which interacts with, and inhibits the function of, NFκB and AP-1 (Ayroldi and Riccardi, 2009) and annexin AI (AnxA1), a calcium-dependent phospholipid binding protein (Perretti and D’Acquisto, 2009). GILZ knockout mice have not been reported, but AnxA1-deficient mice show defective glucocorticoid suppression of inflammation in carrageenin-induced oedema, zymosan-induced peritonitis and antigen-induced arthritis (Hannon et al., 2003; Yang et al., 2004). IL-10-deficient mice develop autoimmune disease and chronic inflammation (Kuhn et al., 1993; Rennick et al., 1995), but effects of glucocorticoids in these mice have not been reported. However, IL-10 has been implicated in negative regulation of corticosterone synthesis, acting at the adrenal gland (Koldzic-Zivanovic et al., 2006), providing a plausible homeostatic mechanism to terminate HPA axis activation once inflammation is resolving. Like IL-10, administration of AnxA1 can mimic a subset of the effects of glucocorticoids (although in T cells, AnxA1 effects may be opposite to those of glucocorticoids) (Perretti and D’Acquisto, 2009). Similarly, ectopic expression of GILZ in T cells (Delfino et al., 2006; Cannarile et al., 2006, 2009) and dendritic cells (Cohen et al., 2006) can mimic some of the effects of glucocorticoid. Indeed, some of the effects of both IL-10 and AnxA1 may even be mediated by GILZ (Cannarile et al., 2009; Yang et al., 2009a), although as IL-10, AnxaA1 and GILZ all alter differentiation or activation state of immune cells (Yona et al., 2004; Cohen et al., 2006; D’Acquisto et al., 2007a,b; Couper et al., 2008; Huggins et al., 2009), such conclusions remain tentative.

Glucocorticoids induce GILZ in a variety of cell types, both immune and non-immune (Clark, 2007), through palindromic GREs in the 5′-flanking region of the GILZ gene (Wang et al., 2004; van der Laan et al., 2008), dependent on dimerisation of GR (Rogatsky et al., 2003). How they regulate IL-10 and AnxA1 remains unclear. *In vitro*, glucocorticoids increase IL-10 expression in monocytes (Mozo et al., 2004), macrophages (Ehrchen et al., 2007) and, dependent on antigen presenting cells, in T cells (Richards et al., 2000), but whether this is related to glucocorticoid-driven alterations in cellular differentiation (see below) or whether glucocorticoid-induced IL-10 indeed mediates some of the effects of glucocorticoids on differentiation as suggested (Richards et al., 2000) remains unknown. Glucocorticoids increase AnxA1 expression on human monocytes and neutrophils *in vivo* (Goulding et al., 1990) and in pituitary folliculostellate cells *in vitro* (Solito et al., 2003) and whilst the underlying mechanisms remain unclear, they appear to involve both non-genomic and genomic actions of glucocorticoids, the latter both modest and relatively slow (Solito et al., 2003). Moreover, glucocorticoids *decrease* AnxA1 mRNA in T cells (D’Acquisto et al., 2008), raising the possibility that glucocorticoid regulation in adaptive and innate immune cells may be related to differentiation/activation state rather than being a direct regulation.

## 5 Context-specific gene regulation by GR

Transcriptional repression by GR has always been the subject of debate, as alluded to above, including the extent to which it is dependent or independent of direct GR DNA binding. However, it is agreed that gene activation requires DNA binding by GR. Much of the early work on GR transcriptional activation was based around a consensus GR binding site, comprising two 6 bp “half sites” arranged in an inverted repeat (palindrome) separated by a 3 bp spacer, derived from comparisons of around 20 GR binding sites in promoters including the MMTV-LTR (Beato et al., 1989; Umesono and Evans, 1989). Subsequent work has confirmed this (Reddy et al., 2009) but additionally shown GR-mediated gene regulation to be much more complex (reviewed in detail in Lefstin and Yamamoto (1998), Grange et al. (2001), Hager et al. (2004), Clark (2007) and Biddie and Hager (2009)). A recent unbiased screen of GR binding sites coupled with transcriptome analysis showed that genes activated by glucocorticoid had GR bound within a median distance of 11 kb from the transcription start site whereas repressed genes had GR bound a median of 146 kb from the transcription start site, suggesting that repression occurs independently of promoter-proximal GR binding (Reddy et al., 2009). *In silico* prediction, genome scanning, chemically directed sequence-specific disruption of GR binding and chromatin immunoprecipitation experiments have shown that sequences that match the GR consensus do not necessarily bind GR in cells (Horie-Inoue et al., 2006; So et al., 2008) and that disruption of GR binding to the conserved half site sequence 5′-WGWWCW-3′ (where W = A/T) only affects a minority of glucocorticoid-regulated genes, both repressed and activated (Muzikar et al., 2009). It has long been clear that many GR binding sites (core sites) are embedded in “composite” glucocorticoid responsive units (e.g. Imai et al. (1990), Alam et al. (1993) and Beato et al. (1996)). Core GR binding sites vary considerably around the consensus (Wang et al., 2004; So et al., 2007) although the precise sequence in and immediately around the core GR binding site in a gene (the glucocorticoid responsive unit) is highly conserved between species (So et al., 2007). Moreover, actual occupancy by GR is influenced by post-translational modification (Blind and Garabedian, 2008) and depends on cell-specific factors (So et al., 2007). Thus, efficient glucocorticoid regulation depends on concomitant binding by other transcription factors at composite elements, to the extent that GR may “tether” at some promoters, retained principally by protein–protein interactions rather than direct interactions with DNA (Lefstin and Yamamoto, 1998). Given that GR homodimerisation, at least of the isolated DNA binding domain, only occurs on DNA binding (Härd et al., 1990; Luisi et al., 1991), then monomers of GR may bind to divergent sequences at composite response elements or by tethering to other transcription factors (Lefstin and Yamamoto, 1998). Thus, the context of the GR binding site is crucial with the outcome – repression, activation or even specificity (MR *vs* GR) – dependent on the cell-specific complement of transcription factors (Grange et al., 1991; Yoshinaga and Yamamoto, 1991; Pearce and Yamamoto, 1993). Whether and how GR contacts DNA might be critical. The GR DNA binding sequence itself acts as an allosteric regulator of GR function, dictating the pattern of regulation that ensues following GR binding (Lefstin and Yamamoto, 1998; van Tilborg et al., 2000; Meijsing et al., 2009). DNA binding induces conformational changes in the dimerisation interface that expose otherwise silent transcriptional activation surfaces (van Tilborg et al., 2000). These conformational changes are exquisitely sensitive to the DNA sequence, with single base pair differences differentially affecting GR conformation and transcriptional regulation (Meijsing et al., 2009). Further complexity is revealed at the level of chromatin, where GR binding is highly dynamic and invariably occurs at either consititutive or hormone inducible nuclease accessible sites (regions of “open” chromatin) at which the requirement for chromatin remodelling complexes differs (John et al., 2008; Biddie and Hager, 2009). These dynamic and gene-specific differences in chromatin remodelling by GR are likely to be highly cell-specific and could underlie the complex kinetics of glucocorticoid responses, where glucocorticoid responsive genes may exhibit alternate activation and repression, with poor correlation in some cases between GR binding to response elements and target gene response (John et al., 2009). Elucidating the nature of GR interactions with target genes, especially in the immune system, will be crucial to understanding their anti-inflammatory effects, but the challenge will be to establish these actions in physiologically relevant settings.

## 6 Glucocorticoid effects on immune cell function

GR is widely, almost ubiquitously, expressed. Thus, glucocorticoids affect virtually all immune cells and, moreover, precise effects depend upon differentiation and activation state of the cell (McEwen et al., 1997), making interpretation of *in vivo* effects in specific cell populations difficult. Nevertheless, several lines of mice with global alteration of GR have provided vital new information about the functions of GR both in regulating the HPA axis and immunity and inflammation. Mice with hypomorphic (*GR*^*hypo*^) or null alleles of GR die neonatally (Cole et al., 1995; Finotto et al., 1999), but haematopoietic progenitors from these mice have been used to reconstitute the immune system in lethally irradiated wild-type mice (Wust et al., 2008). Other models survive and include mice with increased GR density (*yGR* mice with 2 extra GR alleles) (Reichardt et al., 2000), with decreased GR density (*GR*^*+*/−^ mice and anti-sense rat GR transgenic mice, Pepin et al. (1992)) and mice with altered GR function; *GR*^*dim*^ (Reichardt et al., 1998) and *GR*^*M601L*^ mice (Zhang et al., 2009) (a knock-in of a human GR mutant with increased glucocorticoid sensitivity). In all these models the HPA axis is affected because of the central feedback actions of glucocorticoids, compensating for altered GR function in all but the hypomorphic and null mice. All show inflammatory/immune phenotypes (see below). Recent experiments using conditional GR knockout mice as well as transgenic mice have also shed light on the cell-specific functions of GR during immune and inflammatory responses. Tissue-specific models reported include T cell-specific or myeloid cell knockout of GR as well as transgenic mice with thymus over- and under-expression of GR. Some of the immune and inflammatory phenotypes of these various mice have been reviewed in detail elsewhere (Herold et al., 2006; Kleiman and Tuckermann, 2007) and are discussed below.

### 6.1 GR functions in T cells

Glucocorticoid action in T cells and, in particular, in thymus, where naive T cells (that have yet to encounter antigen) develop, has been the subject of intensive research yet remains highly controversial. During T cell development, immature thymocytes progress from double negative (for the CD4 and CD8 T cell markers) to double positive cells (CD4^+^CD8^+^) which undergo positive selection (only thymocytes that bind MHC complexed with self-antigen survive) and negative selection (against cells that interact too strongly with self-antigen) to mature into either CD4^+^ or CD8^+^ single positive cells; the T cell repertoire. Double positive cells, the majority of the thymocyte population, are highly sensitive to glucocorticoid-induced apoptosis (Purton et al., 2004), effective at physiological levels of glucocorticoids (Jaffe, 1924). Much *in vitro* evidence points to a crucial role for glucocorticoids in regulating T cell number, repertoire and function, yet the *in vivo* evidence is discordant. This topic has been extensively reviewed (Ashwell et al., 2000; Godfrey et al., 2001; Jondal et al., 2004; Bommhardt et al., 2004; Herold et al., 2006) and will only be briefly discussed here. Suffice it to say, complete lack of GR globally, in T cells alone or inability to dimerise (*GR*^*dim*^ mice) does not appear to affect thymocyte number or subsets, although these thymocytes are completely glucocorticoid resistant (Reichardt et al., 1998; Purton et al., 2000; Cole et al., 2001; Purton et al., 2002; Brewer et al., 2002) (reviewed in Herold et al. (2006)). Conversely, mice with a global increase in GR levels (*yGR* mice) or function (*GR*^*M604L*^ mice) have normal thymocyte numbers and subsets, yet both lines of mice show increased glucocorticoid sensitivity of thymocytes *in vitro* (Reichardt et al., 2000; Zhang et al., 2009). However, transgenic mice with increased GR in T cells (directed by the proximal *Lck* promoter or a doxycycline-inducible CD2 promoter) show reduced thymic cellularity with increased thymocyte sensitivity to glucocorticoids *in vitro* (Pazirandeh et al., 2002, 2005). Finally, in 2 of 3 models in which GR density is reduced by expression of antisense GR either globally (neurofilament promoter) or in T cells (*Lck* promoter), thymic cellularity is increased, albeit modestly (Pepin et al., 1992; Pazirandeh et al., 2002). However, the 3rd antisense model (also using the proximal *Lck* promoter) showed the opposite effect, with reduced cellularity (King et al., 1995) and altered T cell repertoire (Lu et al., 2000). Reconciling these discrepant results will no doubt inform on the cell-specific roles of GR in thymus and T cell selection.

T cell GR is required to survive lethal activation of T cells. Mice with conditional deletion of GR in T cells (in which the *Lck* promoter was used to drive Cre recombinase-mediated excision of a “floxed” GR gene) showed increased mortality following activation of T cells either by antibody to CD3ɛ or following injection of bacterial superantigen (staphylococcal enterotoxin A), due to failure to repress cyclooxygenase (COX)-2 expression (Brewer et al., 2003). Interestingly, these mice showed much worse tissue damage in the gastrointestinal tract than control mice following T cell activation, although damage in other tissues was similar (Brewer et al., 2003), suggesting a particular role for GR in T cells within the gastrointestinal mucosa in controlling immune activation.

GR is also required in peripheral T cells for the immunosuppressive effects of glucocorticoid therapy in experimental autoimmune encephalomyelitis (EAE; a mouse model of multiple sclerosis) (Wust et al., 2008). Like chimeric mice with *GR*^−/−^ haemotopoietic cells (following lethal irradiation of wild-type mice and reconstitution with *GR*^−/−^ haemotopoietic cell progenitors), *GR*^*LckCre*^ mice (a second line, distinct from the mice mentioned above) showed an earlier onset of disease than controls (indicating a critical role for endogenous glucocorticoid) which was refractory to dexamethasone treatment (Wust et al., 2008). In control mice, but not in *GR*^*LckCre*^ mice, glucocorticoid therapy induced apoptosis of T cells in peripheral lymphoid organs and down-regulated adhesion molecules, thus reducing migration of T cells to the site of inflammation (Wust et al., 2008). Importantly, mice heterozygous for a null mutation of GR (*GR*^*+*/−^ mice), with half the normal level of GR (Wang et al., 2006), showed a more severe EAE disease course with greater inflammation in the spinal cord than control mice and were resistant to the suppressive effects of a sub-maximal dose of dexamethasone (4 mg/kg) that was effective in normal mice (Wust et al., 2008). This suggests that GR density in peripheral T cells is a critical determinant of sensitivity and that despite the presence of functional GR, clinical glucocorticoid resistance can arise. However, in sepsis, another model of inflammation, GR in T cells is not required for glucocorticoid suppression (cited as unpublished data in Kleiman and Tuckermann (2007)).

### 6.2 GR functions in myeloid cells

The phenotype of mice with a conditional deletion of GR in myeloid cells has been reported by 2 groups (Bhattacharyya et al., 2007; Tuckermann et al., 2007; Wust et al., 2008). In both cases, the *LysM* promoter was used to drive Cre recombinase-mediated excision of a floxed GR gene. This results in efficient excision in macrophages and granulocytes in *GR*^*LsyMCre*^ mice, with variable excision in other myeloid cell types including dendritic cells and mast cells (Clausen et al., 1999; Lacy-Hulbert et al., 2007; Tuckermann et al., 2007). Similar to adrenalectomised mice (Bertini et al., 1988), *GR*^*LsyMCre*^ mice showed greater mortality following LPS challenge than control mice (Bhattacharyya et al., 2007). This was attributed to failure to induce DUSP1, required to inhibit p38 MAPK activated by engagement of toll-like receptor (TLR) 4 by LPS (Zhao et al., 2006). However, given that *GR*^*dim*^ mice show normal glucocorticoid induction of DUSP1 in macrophages (Abraham et al., 2006), yet are susceptible to lethal sepsis (cited in Kleiman and Tuckermann (2007)) as unpublished data), it is likely that other important myeloid cell anti-inflammatory actions of glucocorticoids are also required in this model of inflammation. In contrast to endotoxaemia, mice with deletion of GR in myeloid cells remained fully sensitive to therapeutic suppression of EAE by glucocorticoid, although they did show exacerbated disease (Wust et al., 2008).

GR in myeloid cells, but not T cells, are required for glucocorticoid suppression of contact allergy (a T cell-dependent delayed-type hypersensitivity response, such as occurs in response to metals or poison ivy) (Tuckermann et al., 2007). Whereas T cell deletion of GR (*GR*^*LckCre*^) had no effect, deletion of GR in myeloid cells (*GR*^*LysMCre*^) abolished glucocorticoid suppression of inflammation, allowed persistent leukocyte infiltration into the inflamed area and impaired glucocorticoid suppression of macrophage IL-1β, MCP-1 and MIP-2 secretion (Tuckermann et al., 2007). Interestingly, in contrast to irritant-induced skin inflammation (Reichardt et al., 2001), glucocorticoid repression of T cell-dependent contact allergy did not occur in GR^*dim*^ mice (Tuckermann et al., 2007), and although TNFα remained glucocorticoid-suppressible in GR^*dim*^ macrophages, MCP-1 and MIP-2 were not repressed (Tuckermann et al., 2007). Thus, the immunosuppressive effects of GR are likely to result from multiple mechanisms, which are cell-type and stimulus-type dependent.

The elucidation of the cell-specific roles of GR within other leukocyte populations and also within T cell subsets will be informed by future conditional knockouts. Knockout of GR in mast cells, a major target in glucocorticoid suppression of allergic responses (Kassel and Cato, 2002), will be revealing, as will GR disruption in B cells. Like T cells, glucocorticoids also reduce circulating B cell numbers. Consistent with this, blood lymphocyte levels (T and B cells) as well as monocyte/neutrophils (CD11b^+^ cells) were markedly reduced following reconstitution of irradiated wild-type mice with *GR*^*M604L*^ glucocorticoid-hypersensitive haematopoietic progenitor cells (Zhang et al., 2009). Elucidation of the underlying mechanisms may be helpful in the treatment of some early T and B cell leukaemias that respond to glucocorticoids.

## 7 Glucocorticoids alter leukocyte differentiation programmes

As well as profoundly affecting the function of immune cells, glucocorticoids also alter differentiation programmes of progenitor cells (McEwen et al., 1997). Thus, chronically stressful conditions (when endogenous glucocorticoid production is high) or glucocorticoid pharmacotherapy may alter immune cell differentiation and indeed, probably shape the immune response as it develops (Munck et al., 1984; Rook et al., 1994). This may be of relevance in the induction of peripheral tolerance to allergenic stimuli, a major clinical application of glucocorticoids.

Dendritic cells are key antigen presenting cells that bridge the innate and adaptive immune systems. Immature dendritic cells are activated when they capture, process, then present antigens, maturing into immunostimulatory cells in the process. Activated dendritic cells migrate to draining lymph nodes where they interact with naive T cells to instruct the adaptive immune response. Suppression of dendritic cells maturation and function has been implicated in the immunosuppressive effects of glucocorticoids. However, glucocorticoids do not merely suppress dendritic cell activity, but reprogramme them to so-called “tolerogenic dendritic cells” which can elicit a state of hypo-responsiveness in T cells and induce formation of regulatory T (T_reg_) cells (Rea et al., 2000; Rutella and Lemoli, 2004; Chamorro et al., 2009; Luther et al., 2009). Differentiation of tolerogenic dendritic cells is dependent, at least in part, on GILZ (Cohen et al., 2006; Hamdi et al., 2007). Exactly how GILZ mediates anti-inflammatory effects currently remains unclear, although it is likely to depend on protein-protein interactions between GILZ and a variety of intracellular signalling proteins, including the inflammatory regulators NF-κB and activated Ras, the latter being a key protein in signalling to MAP kinases (Ayroldi and Riccardi, 2009). Nevertheless, the active suppression of antigen-specific immunity through T_reg_ induction by glucocorticoid-programmed dendritic cells undoubtedly contributes to their efficacy in allergic disease and probably other chronic inflammatory diseases. Interestingly, as well as promoting a tolerogenic phenotype in dendritic cells, glucocorticoids may also contribute to tolerance through direct effects on T cells. Stimulation of CD4^+^ cells in the presence of glucocorticoid (or even more effectively, together with vitamin D_3_) induced IL-10-secreting T_reg_ cells, able to regulate Th1 responses and autoimmunity (Richards et al., 2000; Hawrylowicz, 2005). A more complete understanding of these effects will improve clinical use of glucocorticoids.

These dramatic effects of glucocorticoids on haematopoietic cell differentiation are not restricted to dendritic cells. Glucocorticoids induce a similar anti-inflammatory phenotype in macrophage differentiation. Work over the past decade has shown that a key mechanism to resolve inflammation is the recognition and phagocytosis of dying cells by monocytes/macrophages (Savill et al., 2002). Glucocorticoids increase macrophage phagocytosis of apoptotic cells, a strongly anti-inflammatory process (Fadok et al., 1998), by at least 2 mechanisms. In already differentiated human monocyte-derived macrophages, they induce a protein S/Mer tyrosine kinase-dependent apoptotic cell clearance pathway (McColl et al., 2009). However, when included during the differentiation process, glucocorticoids also programme blood monocyte differentiation into a highly phagocytic “anti-inflammatory” macrophage phenotype (Giles et al., 2001; Heasman et al., 2003; Ehrchen et al., 2007), a phenomenon that can be modulated by local cytokine environment (Heasman et al., 2003), may be mediated in part by Anxa1 (Maderna et al., 2005) and which also includes induction of Mer tyrosine kinase as well as other anti-inflammatory genes important in phagocytosis, chemotaxis and protection against oxidative stress (Ehrchen et al., 2007). Whether GILZ plays a similar important role in the glucocorticoid-directed macrophage differentiation programme to that it plays in re-programming dendritic cells has not been addressed. Interestingly, these glucocorticoid-programmed macrophages show down-regulation of adhesion-related proteins (Giles et al., 2001; Ehrchen et al., 2007) suggesting they have increased migratory properties. Importantly, in mouse monocytes, glucocorticoids induced a phenotype with low adhesiveness, high migratory capacity and markers suggestive of a tumour-associated macrophage phenotype (Varga et al., 2008), believed to be important for tumour-associated immunosuppression (Sica and Bronte, 2007). The authors further speculate that this may be a mechanism underlying the higher incidence and faster progression of tumours in patients on long-term glucocorticoid therapy (Varga et al., 2008). Intriguingly, in aged mice, elevated plasma glucocorticoid levels were associated with an increase in the proportion of suppressor macrophages in spleen (Kizaki et al., 1998), suggesting that endogenous glucocorticoids may regulate macrophage phenotype *in vivo*, similar to the potent synthetic glucocorticoids used in the *in vitro* studies. However, further clarification of the macrophage phenotypes involved in both the *in vivo* and *in vitro* studies is required before conclusions can be drawn concerning the involvement of glucocorticoids in suppressor macrophage differentiation and function.

## 8 GR agonists; pharmacological and physiological GR activation

A variety of GR ligands are in use clinically which have different potency and differ in their biological efficacy. Different ligands clearly induce different conformations of GR that have different gene regulatory properties (Croxtall et al., 2002; Elmore et al., 2004) - indeed this has recently been exploited in the search for “dissociated” ligands which dissociate the beneficial anti-inflammatory (repressive) effects of GR agonism from the unwanted metabolic (activation) effects (Schacke et al., 2007; De Bosscher and Haegeman, 2009). Structural studies, including of dexamethasone, fluticasone fuorate and, more recently non-steroidal agonists bound in the GR ligand binding domain have shown that GR readily changes conformation to accommodate large moieties on the ligand and have illustrated new possibilities for mode of ligand binding to GR (Madauss et al., 2008; Biggadike et al., 2009). Thus, different conformations of GR resulting from different ligand associations may have cell-specific and target gene-specific properties that offer potential for future pharmacological exploitation.

### 8.1 Endogenous glucocorticoid action

Much of the research on the anti-inflammatory and immunosuppressive actions of glucocorticoids has been carried out with saturating levels of synthetic hormones. In humans, these include prednisolone and methylprednisolone, but in animal models and *in vitro*, dexamethasone is most commonly used. This has provided an over-simplified view of the immunomodulatory actions of glucocorticoids and may have overlooked important opportunities for therapeutic manipulation of endogenous glucocorticoid action. This subject has been previously reviewed (Munck et al., 1984; Wilckens, 1995; Wilckens and De Rijk, 1997; Yeager et al., 2004; Simons, 2008), and the discussion here is restricted to more recent insights.

Synthetic glucocorticoids, especially dexamethasone, have higher affinity, greater bioavailability (unlike the natural hormones, most bind poorly or not at all to corticosteroid binding globulin) and are poorly metabolised, thus they persist in plasma much longer than endogenous glucocorticoids (cortisol, corticosterone). Moreover, the endogenous hormones are released from the adrenal gland in both a circadian and a highly pulsatile (ultradian) manner (reviewed in (Lightman, 2008)). Recent work from the laboratories of Gordon Hager and Stafford Lightman has shown that this pulsatile release of glucocorticoids is coupled to a highly dynamic pattern of GR-mediated transcriptional bursts, driven by rapid recycling of GR occupancy of chromatin binding sites in response to the hormonal pulses *in vivo* as well as *in vitro* (Stavreva et al., 2009). This pulsatility did not occur with constant administration of hormone, nor did it happen with synthetic ligands, including dexamethasone, which failed to cause significant ultradian cycling of GR on chromatin and consequently failed to couple fluctuations in hormone levels with transcriptional response (Stavreva et al., 2009). Thus, transcriptional output can be profoundly altered by synthetic GR ligands or even with natural hormones if not administered in the natural pattern (Stavreva et al., 2009). Moreover, *basal* levels of glucocorticoids *in vivo* exert tonic effects. Thus, macrophages elicited by thioglycollate in the peritoneum of adrenalectomised rats behaved very differently to macrophages from sham operated rats, with much greater TNFα secretion and NO production in the unstimulated state, which could only be marginally increased by LPS/IFNγ stimulation (Lim et al., 2007). Cytokines themselves are potent activators of the HPA axis (Besedovsky and del Rey, 1996; Webster et al., 2002; Sternberg, 2006), and may permanently programme endogenous glucocorticoid secretion when elevated in early life (Shanks et al., 2000). Importantly, when the HPA axis is activated, not only is plasma cortisol elevated (corticosterone in rodents), but so is plasma cortisone (11-dehydrocorticosterone in rodents), itself intrinsically inert due to poor binding to GR, but which is available in plasma (it shows negligible binding to corticosteroid binding globulin) and which can be readily enzymatically converted inside cells to the active steroid by 11β-hydroxysteroid dehydrogenase type 1 (11β-HSD1).

## 9 11β-HSD1 amplifies glucocorticoid action within cells

The last 2 decades have produced a wealth of information on the importance of pre-receptor steroid metabolism. By interconverting active glucocorticoids and inert 11-keto metabolites (cortisone, 11-dehydrocorticosterone), 11β-HSD modulates intracellular access of glucocorticoid to receptors. Type 2 11β-HSD (11β-HSD2) inactivates glucocorticoids *in vivo*, thus protecting the otherwise non-selective MR from occupation by glucocorticoids (Funder, 1997; Seckl, 2000). In contrast, because 11β-HSD1 reactivates glucocorticoids, it increases intracellular glucocorticoid concentration. In addition to cortisone (the natural metabolite), certain synthetic steroids (notably prednisone/prednisolone) are also substrates for the 11β-HSD enzymes. The reaction direction of 11β-HSD1 is dictated by its association with hexose-6-phosphate dehydrogenase (H6PD), which couples glucose-6-phosphate oxidation to NADP reduction, generating NADPH co-factor to drive 11β-HSD1 reductase activity (White et al., 2007; Atanasov et al., 2008). 11β-HSD1 has attracted a lot of recent attention as a potential therapeutic target for metabolic disease, with inhibitors currently under clinical development (Boyle and Kowalski, 2009; Rosenstock et al., 2009). Overexpression of 11β-HSD1 in adipose tissue is associated with obesity in both humans and rodents and in transgenic mice, additionally causes hypertension and insulin resistance (reviewed in Seckl et al. (2004)). Conversely, inhibition of, or deficiency in 11β-HSD1 reduces hyperglycemia and improves insulin sensitivity in non-insulin dependent diabetes in humans and rodents (Seckl et al., 2004). Selective inhibition of 11β-HSD1 also prevented progression of atherosclerosis in *Apoe*^−/−^ mice and lowered levels of circulating MCP-1, a cytokine that recruits monocytes to sites of injury (Hermanowski-Vosatka et al., 2005). It will be important to determine the extent to which these pro-inflammatory effects of 11β-HSD1 are due to its dysregulation in adipose tissue and possibly other tissues in metabolic disease.

11β-HSD1 is widely expressed, with highest expression in classical glucocorticoid target tissues (Whorwood et al., 1992). Given the crucial immunomodulatory actions of glucocorticoids and the strong association between inflammation and insulin resistance/metabolic disease, the role of 11β-HSD1 in the inflammatory response is of great interest. This area has been recently reviewed (Chapman et al., 2006a,b, 2009; Cooper and Stewart, 2009) and the discussion here is restricted to recent developments and questions.

Like GR, 11β-HSD1 is widely expressed in immune cells, but its expression is highly dependent on the differentiation and activation state of the cell. Circulating leukocytes show little or no 11β-HSD1 expression (Thieringer et al., 2001; Gilmour et al., 2006; Fiore et al., 2009), consistent with negligible 11β-HSD1 activity in monocytes (Thieringer et al., 2001) and low levels in lymphocytes (Hennebold et al., 1996) and neutrophils (Kardon et al., 2008). However, following differentiation of monocytes to macrophages (Thieringer et al., 2001) or dendritic cells (Freeman et al., 2005), 11β-HSD1 activity is dramatically increased and is further increased with polarisation to “M1” pro-inflammatory macrophages compared to “M2” anti-inflammatory macrophages (Martinez et al., 2006). Amplification of endogenous glucocorticoids by 11β-HSD1 may influence macrophage state as thioglycollate-elicited peritoneal macrophages from 11β-HSD1-deficient mice showed increased pro-inflammatory cytokine production following LPS stimulation *in vitro*, in the absence of added 11β-HSD1 substrate (Gilmour et al., 2006; Zhang and Daynes, 2007) as well as *in vivo* (Zhang and Daynes, 2007). Interestingly, macrophage 11β-HSD1 is down-regulated following phagocytosis of apoptotic neutrophils; a pro-resolution process (Chapman et al., 2009). The down-regulation of 11β-HSD1 may be part of this mechanism to terminate inflammation. In the absence of 11β-HSD1, mice are more sensitive to endotoxaemia (Zhang and Daynes, 2007), acute inflammation is more severe (Coutinho et al., 2006), and pro-resolution mechanisms may be delayed (Gilmour et al., 2006). We have recently found 11β-HSD1 expression and activity in mast cells, key initiators of acute inflammation and critical targets of the anti-inflammatory actions of glucocorticoids in allergy (Coutinho et al., 2006). Mast cells from 11β-HSD1-deficient mice are hypersensitive to degranulating stimuli (Coutinho et al., 2006), suggesting that endogenous glucocorticoid amplification via 11β-HSD1 restrains their activity. This may be analagous to dendritic cells where it has also been suggested that 11β-HSD1 activity, which is set at high levels in immature dendritic cells and decreases following CD40 activation (a signal that supports cytotoxic T cell differentiation O'Sullivan and Thomas (2002)), may raise the threshold for dendritic cell-induced immune activation, by increasing glucocorticoid action within immature dendritic cells (Freeman et al., 2005).

Within lymphoid cells, 11β-HSD1 activity is present at low levels (Zhang et al., 2005) but is increased following activation of CD4^+^ T cells through the T cell receptor or following polarisation into Th1 or Th2 cells (Zhang et al., 2005), possibly enhancing the ability of endogenous glucocorticoids to suppress Th1 cytokines (in Th1 cells) as well as sparing or promoting Th2 cytokine production by Th2 cells. Thymocytes also express low levels of 11β-HSD1, which is markedly increased, in parallel with GR levels, by burn injury in mice (D’Elia et al., 2009), a strong stimulus to the HPA axis (Hawes et al., 1995). The increase in 11β-HSD1 activity was associated with an increased rate of thymocyte apoptosis (D’Elia et al., 2009). The same may be true in human lymphocyte precursors. In a recent study in acute lymphoblastic leukaemia (ALL) patients, glucocorticoid treatment increased both 11β-HSD1 and GR mRNA levels in glucocorticoid-sensitive leukaemic cells, but decreased 11β-HSD1 mRNA levels in cells resistant to the pro-apoptotic effects of glucocorticoids (Sai et al., 2009). This combination of increased 11β-HSD1 and GR expression is likely to contribute very significantly to the increased glucocorticoid sensitivity of the leukaemic cells, though whether low 11β-HSD1 and GR expression is a cause or an effect of the glucocorticoid resistance in glucocorticoid-resistant leukaemia remains to be determined.

Of great interest is the relevance of 11β-HSD1 to human inflammatory disease. 11β-HSD1 expression is increased in a cell-specific manner by pro-inflammatory cytokines, particularly IL-1 and TNFα (reviewed Chapman et al. (2006a,b, 2009), Cooper and Stewart (2009)) and at sites of inflammation (Ergang et al., 2007; Zbankova et al., 2007; Ahmed et al., 2008; Hardy et al., 2008; Jang et al., 2009). However, it may be decreased in others. Recent data have shown decreased 11β-HSD1 mRNA levels in lung in a mouse model of tuberculosis (Abbott et al., 2009), where, similar to other inflammatory conditions, 11β-HSD1 shows a reciprocal regulation to 11β-HSD2 (Ergang et al., 2007; Abbott et al., 2009). An even further level of complexity is revealed by the findings that in human rheumatoid arthritis, 11β-HSD1 is increased in synovial fibroblasts, whereas synovial macrophages express 11β-HSD2 (Hardy et al., 2008); suggesting a complex control over glucocorticoid availability within the rheumatic joint. 11β-HSD2 is not normally expressed in macrophages (Thieringer et al., 2001; Gilmour et al., 2006), nor is it expressed here during acute inflammation, at least in mice (Thieringer et al., 2001; Gilmour et al., 2006). However, other groups have also reported 11β-HSD2 expression in rheumatoid arthritis patients in synovial macrophages (Schmidt et al., 2005), immortalised B cells (Haas et al., 2006) and peripheral blood mononuclear cells (Olsen et al., 2004). Whether this is part of an adaptive response to inflammation or contributes to glucocorticoid resistance will be important to establish.

Finally, recent work is starting to elucidate the mechanisms that regulate 11β-HSD1 expression. Whilst the C/EBP family of transcription factors (McKnight, 2001; Nerlov, 2007) was implicated some years ago (Williams et al., 2000), recent data have shown the pivotal role of C/EBPβ in the adipocyte expression and tissue-specific regulation of 11β-HSD1 (Gout et al., 2006; Arai et al., 2007; Payne et al., 2007), and importantly, in mediating the effects of pro-inflammatory mediators on transcription of the gene (Ignatova et al., 2009; Yang et al., 2009b). C/EBPβ has also been implicated in the glucocorticoid-induction of 11β-HSD1 in adipose tissue (Sai et al., 2008), although this may be a tissue-specific mechanism (Sai et al., 2008; Yang et al., 2007). Given that C/EBPβ occupies a pivotal position in the integration of metabolic and inflammatory signals (Hu et al., 2007; Millward et al., 2007; Schroeder-Gloeckler et al., 2007; Cho et al., 2008; Staiger et al., 2008; Du and Ding, 2009; Ito et al., 2009; Ruffell et al., 2009), is a critical component of some glucocorticoid responsive units (e.g. Croniger et al. (1997), Savoldi et al. (1997) and Yamada et al. (1999)) and is also a glucocorticoid target gene itself, being regulated at both mRNA and post-translational levels by glucocorticoids in a tissue-specific manner (Gotoh et al., 1997; Kimura et al., 2001; Penner et al., 2002; Berg et al., 2005; Yang et al., 2005a,b), it will be extremely informative to unravel the associations between C/EBPβ, 11β-HSD1 and glucocorticoids in determination of immune cell differentiation and function.

## 10 Summary and conclusions

Many aspects of the anti-inflammatory actions of glucocorticoids have not been covered here. However, it is clear that the field is at an exciting stage. The next few years should provide a big step forward in our understanding of how these important hormones exert their effects, with concomitant advances in the clinical treatment of inflammatory disease.
